# Safety and Blood-Flow Outcomes for Hybrid Recanalization in Symptomatic Refractory Long-Segmental Vertebral Artery Occlusion—Results of a Pilot Study

**DOI:** 10.3389/fneur.2020.00387

**Published:** 2020-05-12

**Authors:** Yan Ma, Bin Yang, Xia Lu, Peng Gao, Liqun Jiao, Feng Ling

**Affiliations:** Department of Neurosurgery, Xuanwu Hospital, Capital Medical University, Beijing, China

**Keywords:** arterial occlusive diseases, hybrid technique, recanalization, vertebral artery, angioplasty

## Abstract

**Objective:** Hybrid recanalization for vertebral artery (VA) long-segmental occlusion using a combination of ostial vertebral endarterectomy and distal endovascular stenting has achieved technical success. The safety and efficacy of the hybrid technique should be further evaluated.

**Methods:** We examined a cohort of refractory patients with long-segmental occlusion in the VA and low flow in the basilar artery (BA). The hybrid technique was performed to achieve the recanalization of VA. Angiograms were analyzed for occlusive length, contralateral VA status and collaterals. Clinical variables, including 30-days outcomes and blood-flow changes within 6 months based on quantitative magnetic resonance angiography (qMRA) with non-invasive optimal vessel analysis (NOVA), were collected pre- and post-operatively.

**Results:** Among 290 consecutive cases with VA initial segment stenosis or occlusion, 14 patients (13 male and 1 female) with symptomatic long-segmental VA occlusion and low flow in the BA were refractory to the best standard medical therapy. The hybrid technique was successful in obtaining recanalization in all but one patient. The mean follow-up period was 17.2 ± 9.2 months. One patient had new ischemic deficits within seven days of the operation. Four patients suffered from transient Horner syndrome postoperatively, but had recovered completely by the 6-months follow-up. Within this period, all revascularization was visible with computed tomography angiography (CTA), and the blood-flow in the BA improved significantly (66.4 ± 15.3 ml/min vs. 104.0±12.9 ml/min, *P* < 0.05) within 6 months. No ischemic events recurred during follow-up.

**Conclusions:** The hybrid technique is potentially a safe and feasible method to achieve recanalization and improve hemodynamic compromise for long-segmental VA occlusion.

## Introduction

Some 20% of patients with posterior circulation ischemia have proximal VA stenosis or occlusion (1). The annual recurrence rate of stroke in patients with symptomatic vertebrobasilar disease is 10–15% (2). In VAST, despite the best medical treatment, patients with more than 50% stenosis still had a 7% recurrence rate of stroke in symptomatic vertebral artery territory during 3-years of follow-up (3). For patients with long-segmental occlusion involving the V1 and V2 segment, there has been little progress in the treatment of patients with recurrent posterior circulation stroke who are refractory to medical therapy. In 2018, we reported the hybrid technique of revascularization for long-segmental occlusion post vertebral artery stenting (4). We now present a pilot study to evaluate the safety and short-term blood-flow outcomes from the hybrid technique performed in our center.

## Methods

### Subjects

From October 2014 to December 2017, 290 consecutive patients with VA initial segment stenosis or occlusion were admitted for ischemic stroke in our center. All patients underwent digital subtraction angiography (DSA) to confirm the lesion. Medical treatment including dual antiplatelet therapy (aspirin 100 mg/d and clopidogre l 75 mg/d for 90 days) and management of risk factors (elevated systolic blood pressure, elevated low-density lipoprotein cholesterol levels, diabetes mellitus, smoking, and excess weight) were prescribed. Advanced treatment was recommended for the patients after consultation with a neurologist, neurosurgeon, and interventional neuro-radiologist. Among these patients, those who experienced (1) chronic long-segmental atherosclerotic occlusion involved in V1 and V2; (2) symptoms such as a focal deficit of bilateral blurred vision, vision loss, dizziness, speech difficulty or numbness or weakness in extremities, refractory to best medical treatment, including recurrent strokes or TIA; (3) failed in simple percutaneous transluminal recanalization were enrolled in our pilot study. After all the necessary evaluations were performed, hybrid recanalization was attempted for these patients.

### Perioperative Imaging Assessment

All patients underwent six-vessel DSA preoperatively to evaluate the status of the vessels including the length of stenosis or occlusion, the contralateral VA stenosis or occlusion; the collaterals from extracranial (ascending cervical artery, deep cervical artery, and muscular branches at the level of C1 or C2) and intracranial system (posterior communicating artery) were recorded. Magnetic resonance imaging (MRI) was performed pre- and postoperatively to evaluate ischemic lesions by using a 3.0-T system (GE Medical Systems, Milwaukee, Wisconsin, USA). The imaging protocol included T1, T2-weighted spin-echo and diffusion-weighted imaging series. The slice thickness was 5 mm and the intersection gap was 1.6 mm. All the data were assessed by an experienced board-certified neuro-radiologist. BA blood flow changes resulting from the disease process and at 6 months after the recanalization surgery were measured using quantitative magnetic resonance angiography with non-invasive optimal vessel analysis (NOVA, VasSol, Inc., Chicago, Illinois).

### Clinical Assessment and Follow-Up

All patients were professionally assessed by at least one senior neurologist and one senior neurosurgeon. Neurologic status was evaluated pre-procedure, and at seven and 30 days postoperatively using the National Institutes of Health Stroke Scale (NIHSS). Postoperative complications (including Horner syndrome, cranial nerve injury and lymphatic injury) were recorded for every patient. The same assessments were performed at 6 and 12 months follow-ups in the outpatient department.

### Hybrid Technique in Revascularization

**Figure 1:**
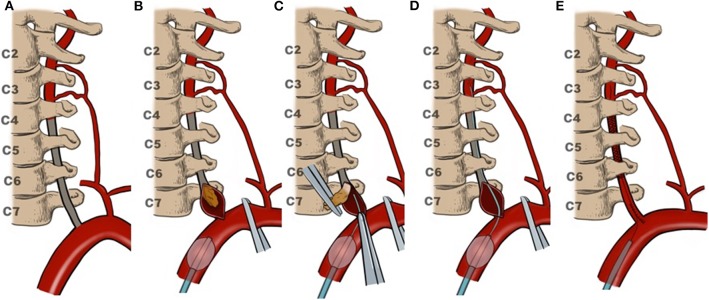
Illustration of VA recanalization. **(A)** Occlusion involved the V1and V2 segment; **(B)** endarterectomy for V1segment first; **(C)** delivering microcatheter and microwire along with the interface between the plaque/thrombus and vessel wall; **(D)** microcatheter angiography confirming the distal VA real lumen; **(E)** closing V1 incision then stenting for the V2 segment.

The hybrid procedure was originally reported by Yang et al. (4) After V1 segment exposure (5) and systemic heparinization, an 8-French balloon guide catheter (Concentric Medical Inc., Mountain View, California, USA) was placed in the proximal SCA through the femoral artery approach and then the balloon was expanded to achieve temporary occlusion. The V1 segment endarterectomy was performed first (5). Through the guide catheter, a 0.014-inch microwire (Pilot, Abbott Vascular, Jamaica, New York, USA) with a microcatheter (Echelon, eV3 Inc., Plymouth, Massachusetts, USA) was delivered, and with the help of a microscope they were inserted into the distal lumen manually along the interface between the plaque/thrombus and vessel wall. Then microcatheter angiography was performed to confirm the distal VA real lumen. After the microwire was left in the distal VA, continuous suture was performed on the V1 segment. Along the microwire left in real lumen, distal occlusion could be recanalized by the balloon-mounted stent (Apollo, MicroPort, Shanghai, China) (see illustration in Figures 1A–E).

### Statistical Analysis

Statistical analysis was performed using SPSS 21.0 (SPSS Inc., Chicago, IL, USA). *T*-test analyses were performed for paired samples. A *P* < 0.05 was considered statistically significant.

## Results

### Clinical Evaluation

Of 290 consecutive patients with VA origin lesions, 14 refractory patients (4.8%) were enrolled in our study (1 woman and 13 men). The mean age was 63.3 ± 7.4 years (range, 53–77 years). Median NIHSS at presentation was 1.6±0.7 (range, 1–3). All patients underwent attempted hybrid recanalization of the proximal (V1+V2) segment of VA, which was successful in all but one procedure, resulting in a technical success rate of 92.9%. The mean operation duration was 4.4 ± 1.1 h. There were no cases of perioperative death. One patient (7.1%) suffered from new ischemic neurologic deficits within 7-days post-procedure, and four patients (28.6%) suffered from transient Horner syndrome, but all resolved within 6 months. All patients were followed-up in the outpatient department according to the protocol, with a mean follow-up period of 17.2 ± 9.2 months. No death or recurrent stroke occurred during the regular follow-up period. The clinical characteristics and risk factors are summarized in Table 1.

**Table 1:** Clinical and angiographic characters.

**Patient number**	**Age (years)**	**Sex**	**Hypertension**	**Diabetes**	**Smoking history**	**Criminal VA (R/L)**	**Occlusive length (mm)**	**Contralateral VA (occl/dys)**	**Collaterals**			**Qualifying events (location)**	**NIHSS**
									**Ascending cervical artery**	**Muscular branches at C1 or C2**	**PCA**		
1	75	M	+	+	+	L	55	dys	+	+	+	Stroke(cerebellum)	1
2	58	M	+	−	+	R	44	occl	+	−	+	TIA	2
3	59	M	+	+	−	R	45	occl	+	−	+	TIA	1
4	53	M	+	+	−	R	62	occl	+	−	+	TIA	1
5	57	F	−	−	+	R	91	occl	+	+	+	Stroke (cerebellum temporal lobe)	3
6	63	M	−	+	+	L	27	occl	+	+	+	Stroke (temporal lobe)	1
7	61	M	−	−	+	L	74	occl	+	+	+	TIA	2
8	72	M	−	−	−	R	45	dys	+	−	+	Stroke(cerebellum)	3
9	70	M	+	−	+	R	42	occl	+	+	−	Stroke (occipital lobe)	1
10	55	M	−	−	−	R	73	occl	+	+	−	Stroke (temporal–occipital junction)	2
11	77	M	+	+	−	R	53	occl	+	+	+	Stroke (temporal–occipital junction)	1
12	61	M	+	−	+	R	103	dys	+	+	+	Stroke (cerebellum)	1
13	62	M	+	+	+	R	57	occl	+	−	−	Stroke (cerebellum thalamus)	2
14	63	M	+	−	−	R	59	dys	+	−	+	Stroke (cerebellum thalamus occipital lobe)	2

*M, male; R, right; L, left; M, male; F, female; +, positive; – negative; occl, occlusion; dys, dysplasia; PCA, posterior communicating artery; NIHSS, National Institutes of Health Stroke Scale*.

### Angiographic Evaluation

The target VA included eleven cases on the right side and three on the left. The contralateral VA demonstrated occlusion in 10 cases and dysplasia in the others. The mean occlusive length was s 59.3 ± 20.3 mm (range, 27–103 mm). All patients had collaterals from ascending cervical artery to the V2 segment through muscular branches. Eight patients (57.1%) had compensatory blood-flow from the muscular branches at the level of C1 or C2, and 11 cases (78.6%) from a patent posterior communicating artery (see Table 1). Within the follow-up period, all revascularization was absent of restenosis (defined as >50% luminal narrowing), as confirmed by computed tomography angiography.

### NOVA Blood-Flow Analysis

**Figure 2:**
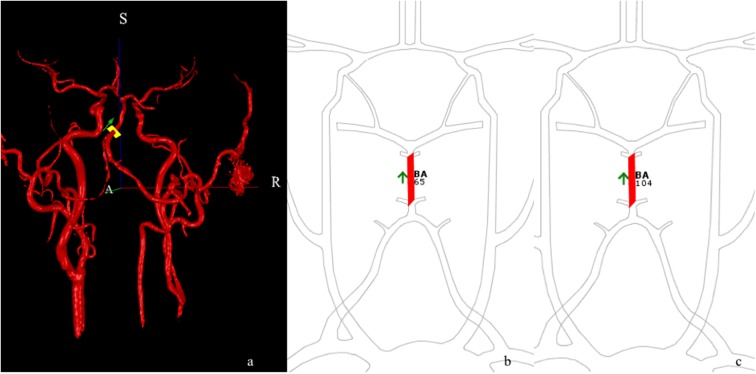
qMRA in an R-VA long-segmental occlusion and left VA dysplasia patient after right-side revascularization. Three-dimensional angio-image **(a)** with ROI (yellow cross-section) placed on the BA. S and R represent superior and right, respectively. qMRA volumetric maps show the increase in blood flow from a preoperative BA of 65 mL/min **(b)** to postoperative BA of 104 mL/min **(c)** within 30 days of revascularization.

**Figure 3:**
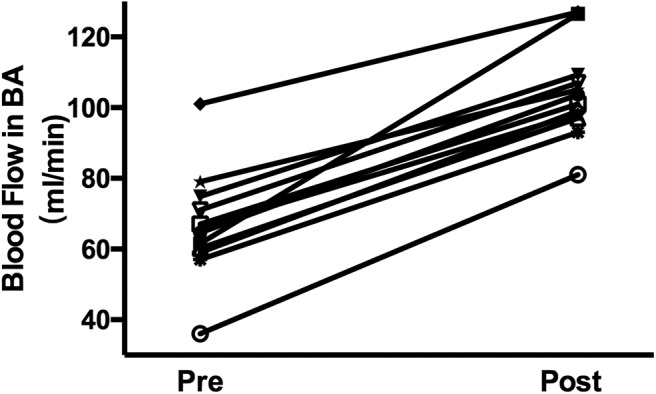
Blood flow changes in the BA of the 14 patients pre- and post-revascularization.

Figure 2 shows an example of 3-D MRA images of the BA with blood-flow measurements. Blood-flow changes in the BA of the 14 patients were summarized as shown in Figure 3. Blood-flow in the BA significantly increased between pre- and post-operative measurements (66.4 ± 15.3 ml/min vs. 104.0 ± 12.9 ml/min, *P* < 0.05).

### Case Illustration

**Figure 4:**
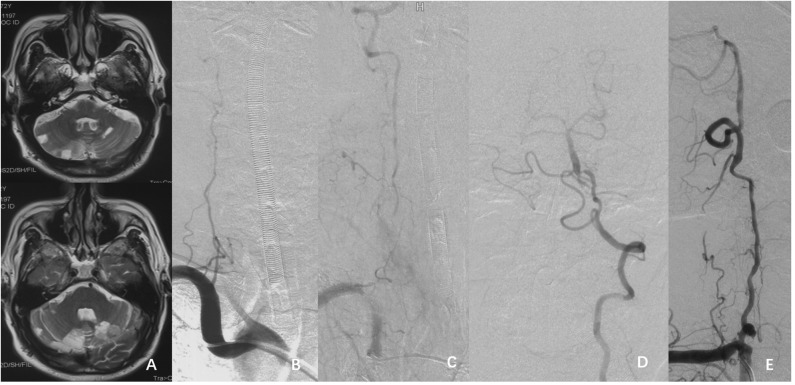
**(A)** MRI T2 weighted image showing multiple infarctions in the bilateral cerebellar hemispheres. **(B–D)** DSA showing the right VA occlusion and the dysplasia in the left V4 segment. **(E)** Follow-up DSA showed the patency of right VA 1-year postoperatively.

A 72-year-old man presented with paroxysmal vertigo and nausea. The symptoms were aggravated, with unstable walking, despite treatment with the best medicine. MRI showed multiple infarctions located in bilateral cerebellar hemispheres (Figure 4A). DSA demonstrated right VA occlusion and left VA terminal segment dysplasia (Figures 4B–D). He underwent hybrid recanalization for the right VA, and the one-year postoperative DSA (Figure 4E) showed persistent patency.

## Discussion

The VA initial segment is a site commonly affected by atherosclerotic stenosis. 20% of patients with symptoms of posterior circulation ischemia have proximal VA stenosis or occlusion (1). Symptomatic long-segmental occlusion of V1 and V2 segments being refractory to the best medical treatment remains a challenging management issue. Neither primary endarterectomy nor interventional therapy can routinely achieve revascularization (6, 7). In recent decades, hybrid techniques combining open surgery and interventional treatment have demonstrated special advantages in recanalization for long-segmental occlusive lesions (8–10). For this complex occlusive vascular disease, direct endarterectomy addresses the plaque and part of the thrombus in the V1 segment to obtain revascularization of the VA ostium, while also directly revealing the interface between the plaque and vessel wall. Along with this interface, a microwire can easily be inserted into the distal true lumen in order to allow distal revascularization by stenting.

In this pilot study for revascularization of VA occlusion, there were satisfactory technique success rates and relatively low complication rates. These might benefit from preoperative evaluations including hemodynamics and careful evaluation of angiographic anatomy. Regarding the indications for revascularization of long-segmental VA occlusion, hypoperfusion is an important ischemic mechanism in posterior circulation occlusion (11). It may also reduce the wash-out of emboli and prompt local thrombus formation (12–14). In our study, all the patients had symptomatic VA occlusion that was refractory to the best medical therapy, and preoperative blood-flow analysis by NOVA demonstrated low flow in the BA (15). Furthermore, the patients suffered from occlusion that was restricted to the V1 and V2 segments, which made recanalization feasible. The postoperative blood-flow analysis revealed an increase in blood-flow in the BA, confirming the successful reversal of hypoperfusion.

An important consideration in planning the procedure relates to protection of the collaterals to the proximal VA. In our cohort, muscular branches from the ascending cervical artery to the V2 segment were the most common collaterals disclosed by preoperative DSA. The protection of the collaterals is one of the key points in achieving a decrease in perioperative strokes, and is also an advantage of the hybrid technique, which achieves revascularization in the V2 segment by interventional methods. This avoids the risk of damage to the V2 collaterals that would be induced by direct open surgery of the transverse foramen.

In the pilot study, the hybrid procedure failed in one patient because of distal dissection. One patient suffered from symptomatic ischemic stroke, and MRI revealed new focal infarction in the cerebellar hemisphere. Four patients suffered from temporary Horner syndrome, but all the symptoms were resolved within 6 months. Horner syndrome is the most common complication (18.2–100%) for VA endarterectomy and transposition (4, 16, 17), but most incidents are temporary and resolve completely. No cranial nerve and thoracic duct injuries occurred in this cohort, which might be due to having more cases on the right side, and bilateral anatomical differences.

## Limitations

In this pilot study, only cases of chronic long-segmental atherosclerotic occlusion involving V1 and proximal V2 with recurrent ischemic symptoms that failed to respond to simple interventional therapy were enrolled. Hybrid recanalization was not utilized in longer occlusive lesions, such as those extending to V3 or V4. This pilot study also has some limitations owing to its non-randomized, un-blinded, single-center basis, and small sample size. In addition, the follow-up period was not of a sufficient duration, and hemodynamics status was not one of the inclusion criteria. As a result, these limitations reflect the necessity for further research in this field.

## Conclusion

The hybrid technique is, potentially, a safe and feasible method to achieve revascularization and improve hemodynamic compromise for long-segmental proximal VA occlusion. Clinical trials with a larger sample-size and long-term follow-up are necessary to confirm these findings further.

## Data Availability Statement

The raw data supporting the conclusions of this article will be made available by the authors, without undue reservation, to any qualified researcher.

## Ethics Statement

The research protocol was reviewed and approved by the ethics committee of Xuanwu Hospital, Capital Medical University, to perform analysis and publish de-identified data. All subjects gave written informed consent in accordance with the Declaration of Helsinki. The patients approved publication of de-identified data for academic communication.

## Disclosure

The authors report no conflict of interest concerning the materials or methods used in this study or the findings specified in this paper.

## Author Contributions

YM: contributed to the preparation of the manuscript, data collection, and statistical analysis. LJ and FL: conceived and designed the research. BY, XL, and PG: carried out data review and interpretation. All authors final approval of the version to be published.

## Conflict of Interest

The authors declare that the research was conducted in the absence of any commercial or financial relationships that could be construed as a potential conflict of interest.

## Abbreviations

VAST: vertebral artery stenting trial
DSA: digital subtraction angiography
ICA: internal carotid artery
IJV: internal jugular vein
SCA: subclavian artery
SM: sternocleidomastoid muscle
TIAs: transient ischemic attacks
VA: vertebral artery
BA: basilar artery
CTA: computed tomography angiography
qMRA: quantitative magnetic resonance angiography
NOVA: noninvasive optimal vessel analysis.
